# Time-series dataset of air temperature, fruit surface temperature and humidity in commercial apple cold storage

**DOI:** 10.1016/j.dib.2026.113157

**Published:** 2026-08-10

**Authors:** Reiner Jedermann, Tuany Gabriela Hoffmann, Daniel Alexandre Neuwald, Felix Büchele, Pramod Vasantrao Mahajan

**Affiliations:** a University Bremen, Institute for Microsensors, -Actuators and -Systems (IMSAS), Otto-Hahn-Allee 1, 28205 Bremen, Germany; b Department of Systems Process Engineering, Leibniz Institute for Agricultural Engineering and Bioeconomy (ATB), Max-Eyth-Allee 100, 14469 Potsdam, Germany; c Lake of Constance Research Center for Fruit Cultivation (KOB), Schuhmacherhof 6, 88213 Ravensburg, Germany; d Department of Production Systems of Horticultural Crops, University of Hohenheim, Emil-Wolff-Str. 23, 70599 Stuttgart, Germany

**Keywords:** Dew point, Surface temperature, Postharvest, Storage conditions, Temperature mapping, Refrigeration control, IoT sensors

## Abstract

Temperature and humidity strongly influence fruit quality during cold storage. Temperature should be maintained slightly above the freezing point for most fruits, and humidity should be maintained in equilibrium with fruit’s moisture to reduce mass loss, while avoiding condensation on its surface. Besides absolute temperature, the difference between fruit surface and air temperature plays a key role for phase change phenomena such as condensation, evaporation, mass loss, and heat transfer by cooling air. Whereas spatial air temperature distribution in the postharvest cold chain has been covered by several studies, data on humidity distribution are scarce. This time series dataset provides multi-point measurements of fruit surface and air temperature, and air humidity recorded with IoT sensors inside a commercial apple cold room. The changes in these parameters across different locations and storage periods can be used to identify condensation spots and to create a better understanding of cooling processes under high humidity conditions and their optimization.

Specifications Table

**Table:** 

Subject	*Agricultural Sciences - Food Science - Food Technology*.
Specific subject area	*Local and temporal variation of temperature, humidity, and dew point during cold storage of fruit*
Type of data	Tables with measurement data (Tab separated text files) Additional tables with sensor locations, specific test conditions, events, and room geometry MATLAB example code for plotting
Data collection	The temperature difference was calculated from pairs of digital chip sensors. Sensirion STS35 sensors were applied to measure fruit surface temperature, Sensirion SHT45 sensors were applied for air temperature and for relative air humidity. The tests were carried out in a cold room containing 102 - 157 apple bins (∼300 kg each, cultivar Pinova). Between 10 and 17 bins were equipped with sensor pairs per test. Sensor accuracy was improved by calibration under constant temperature and humidity conditions (1.5 °C, 98.5%RH) resulting in an average error of ± 0.03 °C for temperature difference per sensor pair. Humidity sensors had a total error of ±1%RH, including sensor drift over time under high humidity conditions.
Data source location	Data were recorded at Lake of Constance Research Center for Fruit Cultivation (KOB), Ravensburg, Germany
Data accessibility	Repository name: Mendeley Data Data identification number: doi:10.17632/h4sghvyjyt.2 Direct URL to data: https://data.mendeley.com/datasets/h4sghvyjyt/2
Related research article	[1] R. Jedermann, T.G. Hoffmann, D.A. Neuwald, F. Büchele, P.V. Mahajan, Dew point undershoot mechanisms driving surface condensation in commercial apple cold storage, Postharvest Biology and Technology, 236(2026). https://doi.org/10.1016/j.postharvbio.2026.114264*.*

## 1 Value of the Data

• Food quality highly depends on the cumulative temperature and humidity history of the product. High-resolution measurements of fruit surface temperature, air temperature, and humidity within stacked bins of apples in cold rooms enable quantitative assessment of storage performance, facilitate early detection of suboptimal conditions, and support evidence-based optimization of process parameters to preserve product quality.
• Temperature and humidity in cold rooms are marked by high variations across different locations and over time due to airflow patterns, door openings, product stacking geometry, and refrigeration cycles. Such variability generates distinct microclimates, leading to non-uniform cooling rates and differential moisture exchange within the same batch. For example, fruit located near doors or evaporators may experience fluctuating conditions compared to bins positioned deep within pallet stacks, resulting in heterogeneous storage conditions.
• In particular, the complexity and limited empirical data available regarding localized humidity dynamics and surface condensation events, which occur when fruit surface temperature falls below the ambient air dew point, highlight the importance of the data. These transient phenomena are difficult to capture but are critically important, as surface wetness promotes microbial growth. Systematic measurements of fruit surface temperature in relation to dew point provide novel insight into the frequency, duration, and spatial distribution of condensation events under commercial storage conditions.
• The data set gives first insight into the effect of modified storage conditions, such as temperature setpoint and ventilation on/off periods, on temperature and humidity distribution and formation of critical condensing conditions.
• The dataset provides a robust experimental basis for the development and validation of predictive models describing spatial temperature and humidity distribution, condensation risk, moisture migration, and associated storage disorders. By integrating fruit surface temperature with air temperature and humidity measurements, the data enables mechanistic modelling of heat and mass transfer processes, supporting improved simulation of storage scenarios and risk-based cold chain management strategies.
• Researchers can utilize the data for training and testing of machine learning methods and verification of computational fluid dynamics (CFD) simulations and digital twin models of fruit storage facilities.

## 2 Background

Previous tests [2] verified that condensation can be observed during commercial apple cold storage. This first test was limited to few positions in door side bins and the storage phase after achieving stable temperature conditions. Moisture starts to condense on the fruit surface when the surface temperature falls below the dew point, so called dew point undershoots (DPU). A series of new tests was started in autumn 2024 to provide additional data for locations deeper in the fruit stack and to cover different storage phases. The goal of the new tests was to determine when (i.e., during which storage phases), where (i.e., at which spatial locations) and how (i.e., by which mechanisms) DPU events occur.

This data set provides detailed measurements for each location and documentation of all setpoints and ventilation changes. Researchers can relate observed temperature and humidity fluctuations in different locations and storage periods to data on cooling conditions and further analyse the data regarding local efficiency of cooling, and prediction of condensation risks.

## 3 Data Description

The data set contains the full set of measurements applied in [1], except test T1, which was carried out by another author team [2]. All data are available in a single zip file (TTH_ColdRoom_June19.zip) on Mendeley Data [3].

The setup folder contains the main metadata files required for reuse of the dataset, including room geometry, and general experimental parameters. Test-specific metadata are provided in the individual test folders and include sensor-position lists, test conditions, and event logs documenting changes in setpoints, ventilation settings, defrosting, loading, and other relevant operational events. Together, these files allow the raw sensor measurements to be linked to sensor location, calibration information, timestamps, and cold-room operating conditions.

Table 1 gives an overview of the different conditions applied in the four reported tests T2 – T5.

**Table 1:** Overview of test conditions.

Parameter	Values range
Test Number	T2 – T5
Date	September 2024 to April 2025
Duration	4 days to 4 weeks
Total number of bins	102 to 157
Sensors	10 to 17 locations in apple bins by measuring with wireless and wired sensors, with additional sensors for supply and return air
Storage phase	Cool-down / stable conditions / end of storage season
Atmosphere conditions	No control (normal air) / controlled atmosphere (CA, 1 kPa O_2_ and 2.5 kPa CO_2_)
Room / Refrigerant	Room 4 with propane (R290) or room 3 with hydrofluorocarbon (R134a)
Temperature setpoint	Set point limits between 0.5 °C – 1.5 °C and 1 °C – 2.6 °C
Defrosting	Always 4 defrosting cycles per day (at 1:45, 7:45, 13:45, 19:45). By default, defrosting was assisted by ventilation only. Up to 3 cycles can be replaced by electrical defrosting.
Ventilation	Ventilation off period after cooling (40 to 80 min) and ventilation on period (20 to 40 min), Pre-ventilation before cooling starts (2 to 15 min)
Remarks	List of further specific conditions for individual tests, e.g., high initial temperature variance, a partly filled room, or cooling interruptions

The data set is structured in subfolders (Table 2) for measurements and specific test conditions (Folders t2 – t5), general test setup including room geometry and calibration offsets (Folder setup) and example MATLAB scripts for plotting (Folder MATLAB).

**Table 2:** Files in zip archive, ‘*’ = test number, ‘#’ = sensor name (4 to 15 sensors per set).

Tests	Folder	Filename pattern	Content
General	setup	CalibrationOffsets.txt	Measured calibration offsets per sensor
General	setup	GeometricalParameters.docx	Dimensions of cold room, bins, and gaps between bins
*=2…5	t*	t*._Conditions.docx	Specific test conditions
*=2…5	t*	t*._Events.docx	Events and modification of cooling parameters
*=2…5	t*	t*._Events.txt	Events in txt format including Unix timestamps
*=2…5	t*	t*._SensorList.txt	Sensor positions
*=2…5	t*	t*.Lora#.txt	Sensor measurements
*=2…5	t*	t*.Gas.txt	Measurements of CO_2_ and O_2_
*=2,3	t*	t*.Nuc_#.txt	Measurements of additional wired sensors
T3	t3	t3.Asl_#.txt	Measurements of air speed loggers
*=2…5	MATLAB	plotAll_T*.m	Plot all sensors from one data set
*=2…5	MATLAB	plotCombined_T*.m	Combine different sensors, e.g. to reproduce figures in [1]
*=2…5	MATLAB	plotGas_T*.m	Plot CO_2_ and O_2_ measurements
T3	MATLAB	plotASL_T3.m	Plot air speed

### 3.1 Setpoint and ventilation settings

Events, such as setpoint and ventilation changes are listed in the “t*._Events.docx” tables. See Table 3 for example. The column “Fan” gives the ventilation setting, e.g., “40/20 Pre 2″, where the first number gives the duration of the off period in minutes (40), followed by the duration of the on period (20). The additional parameter “Pre” gives the ventilation time in min before cooling starts (2).

**Table 3:** Example of an event list.

Date	Remarks	Setpoint [ °C]	Fan [min off/on]	Electrical defrosting [min heat/drip]
2025–03–07 12:00	Start loading, several door openings	0.5 – 1.5	40/20 Pre 2	1 × 10/10
2025–03–08 10:00	Loading of bins completed	–	–	–
2025–03–10 10:00	Increase set point	1.5 – 2.5	–	no

The default setting is 4 defrosting cycles per day having 30 min ventilation followed by 10 min dripping phase without ventilation. Replacement of these cycles by electrical defrosting is indicated in the last column, e.g. “1 × 30/10″. The first value gives the number of replaced cycles per day (1), followed by the duration of the heating (30) and dripping (10) phases in min.

The “t*._Events.txt” tables contain the same data in tab separated text format. Additional columns contain the timestamp in Unix format (seconds since 1970) and as days since the test start. It is strongly recommended to use only these timestamp formats for further data analysis. The timestamps in string format are only for better readability but can be misinterpreted due to switching between day-light-saving offsets. The last three column contain the number of electrical defrosting cycles per day, their duration of heating in min and dripping time.

### 3.2 Sensor positions

**Fig. 1:**
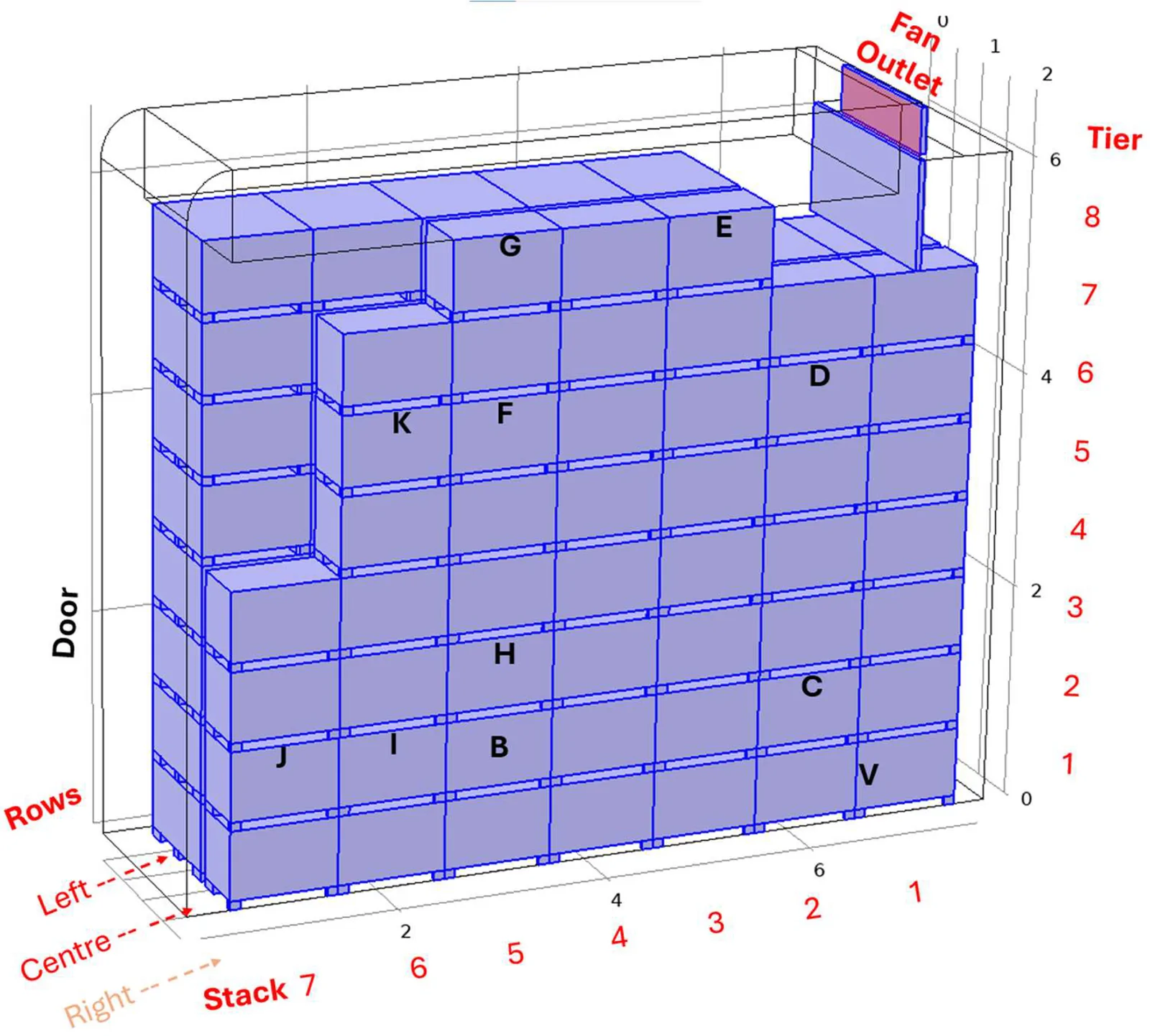
Layout of apple storage room showing numbering of rows, stacks, and tiers. Only left half of cold room was plotted for better visibility. Letters indicate sensor positions during test T2. Dimensions are in meter.

The cold room contains 3 rows of 7 stacks with 8 tiers in height of bins. Some positions under the evaporator and at the door side remain empty (Fig. 1). Most sensors were placed in the middle row.

**Fig. 2:**
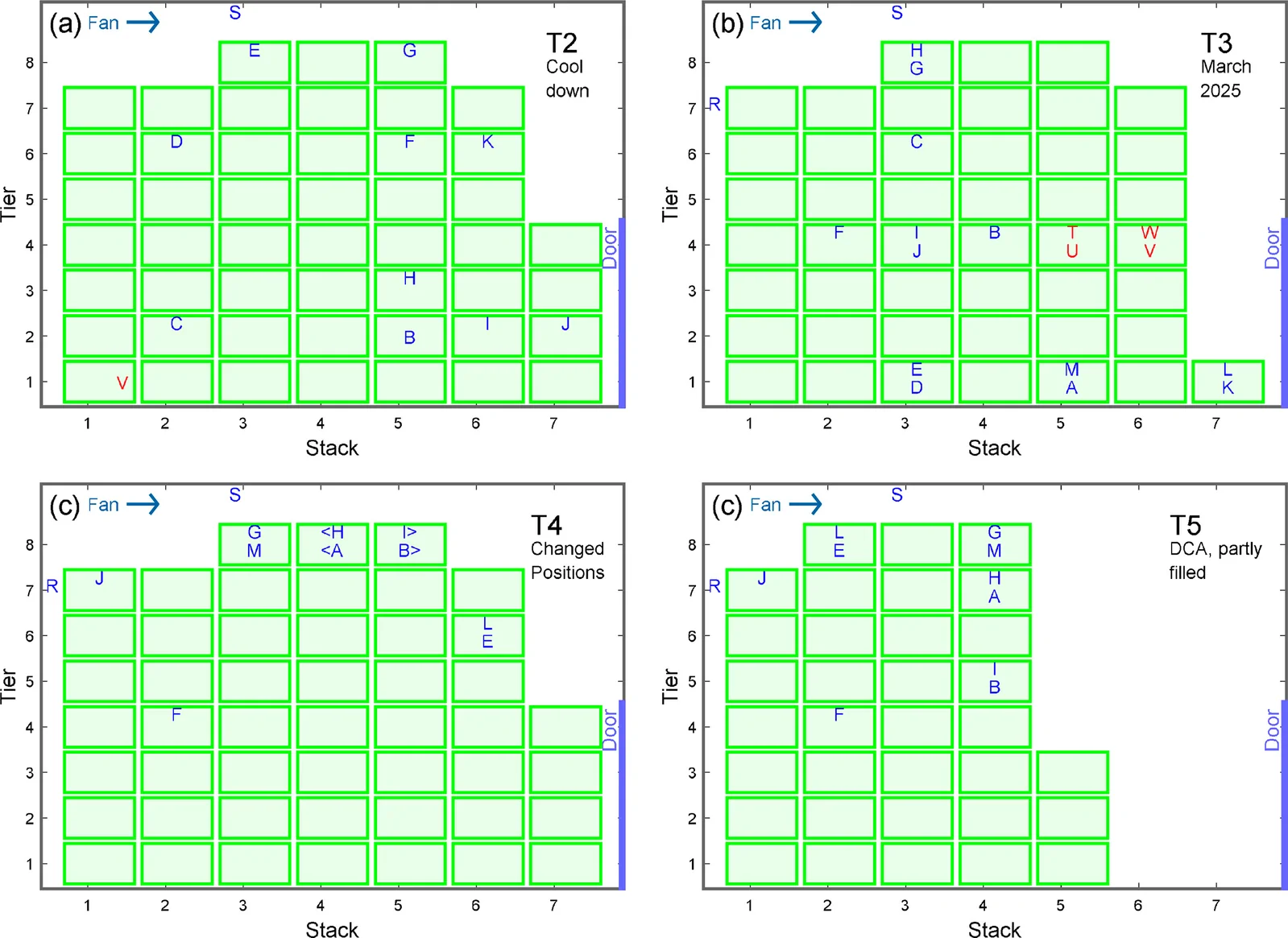
Sensor positions in the middle row and stacking height for T2 – T5. Left and right row have the same stacking height, except for door side stacks 7 and 8 which were filled up to tier 8. Sensor positions in left and right row are indicated by “<” and “>”. Drawing not to scale. Red letters indicate wired sensors.

The sensor positions were changed for each test (Fig. 2), as listed in the “t*._SensorList.txt” files. The columns contain the sensor name, stack, tier, and position inside the bin (t = top horizontal centre, h = half height, l = left row, r = right row).

### 3.3 Sensor data

All sensor data are reported in tab separated text files. The first column gives the timestamp in string format, followed by the Unix format. Measurements are reported in the order - air temperature, surface temperature in °C and relative humidity in %. Gas sensors report CO_2_ and O_2_ concentrations in %, and air speed loggers (ASL) report the speed in m/s.

The default sampling rate was 150 s during T2 and 60 s during the subsequent tests. The actual sampling rate deviated due to inaccuracies of the micro controller’s low power clock. The additional wired sensors had a sampling rate of 30 s.

Additional climate data for ambient temperature can be retrieved from https://www.wetter-bw.de/Internet/AM/NotesBwAM.nsf/bwweb/Bavendorf .

### 3.4 MATLAB scripts

The MATLAB folder contains scripts for a first visualisation of the data. The scripts calculate the dew point according to [4] for easier comparison with surface temperature. The “plotCombined_T*.m” scripts can be used to reproduce figures in [1], different graphs can be selected by changing the “PLOT_NUM” constant. Setpoint changes are marked by dotted lines.

**Fig. 3:**
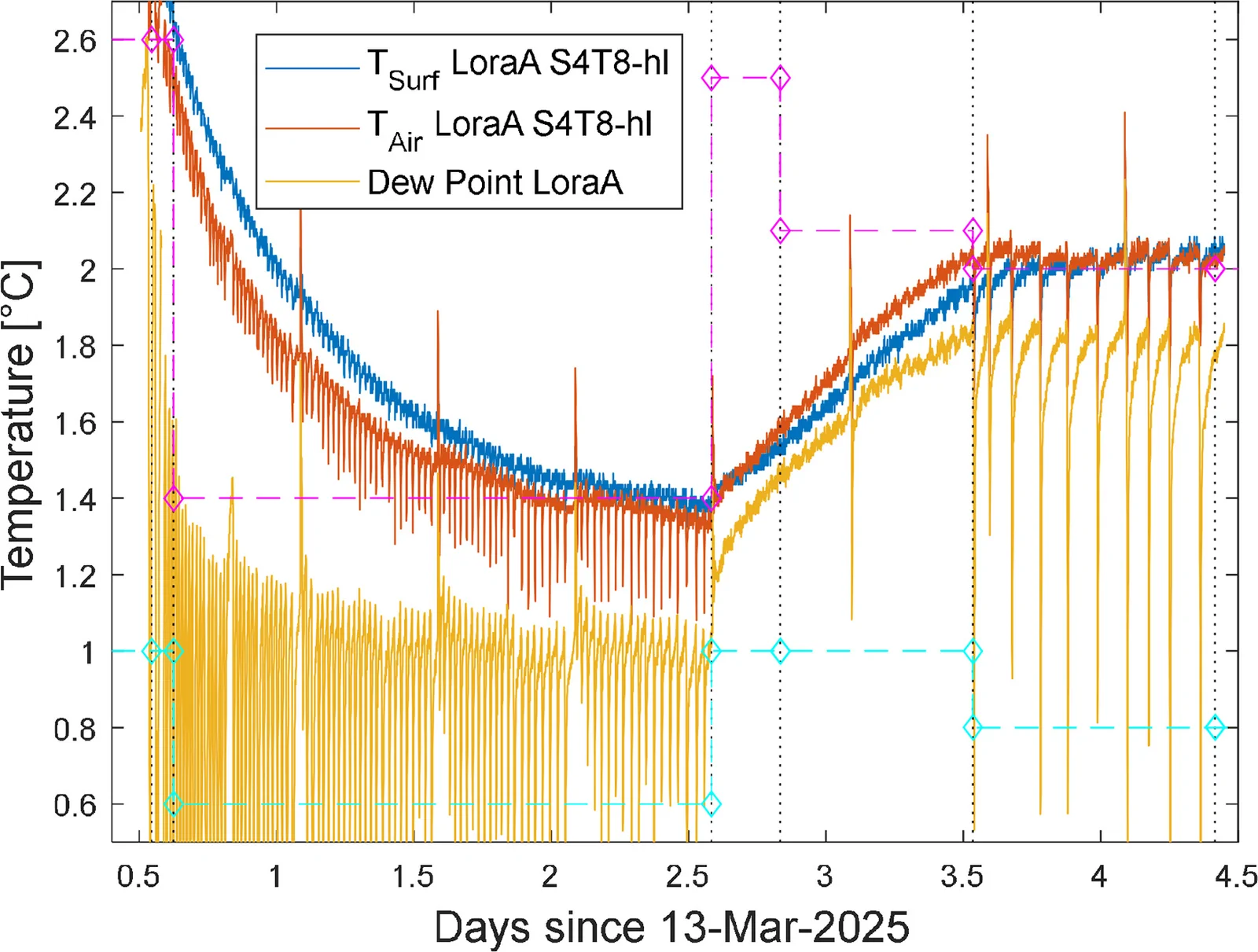
Air, surface, and dew point temperature for sensor LoraA in test T4. The direction of the air-surface temperature difference changes after increasing the setpoint. Dew point comes close to surface temperature. Lower and upper setpoints are marked by dotted lines.

**Fig. 4:**
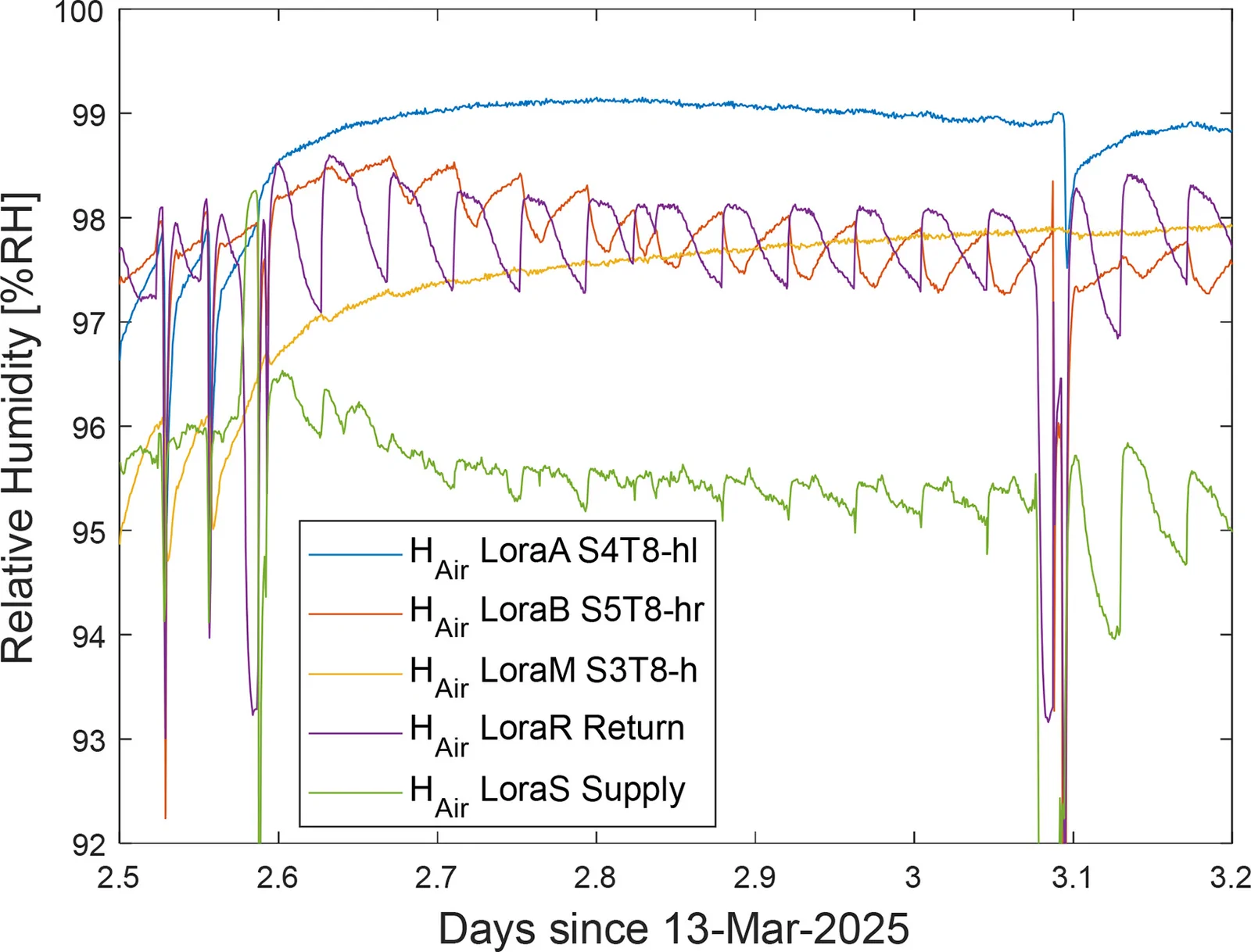
Humidity changes in various positions during test T4. Drops in relative humidity by cooling.

**Fig. 5:**
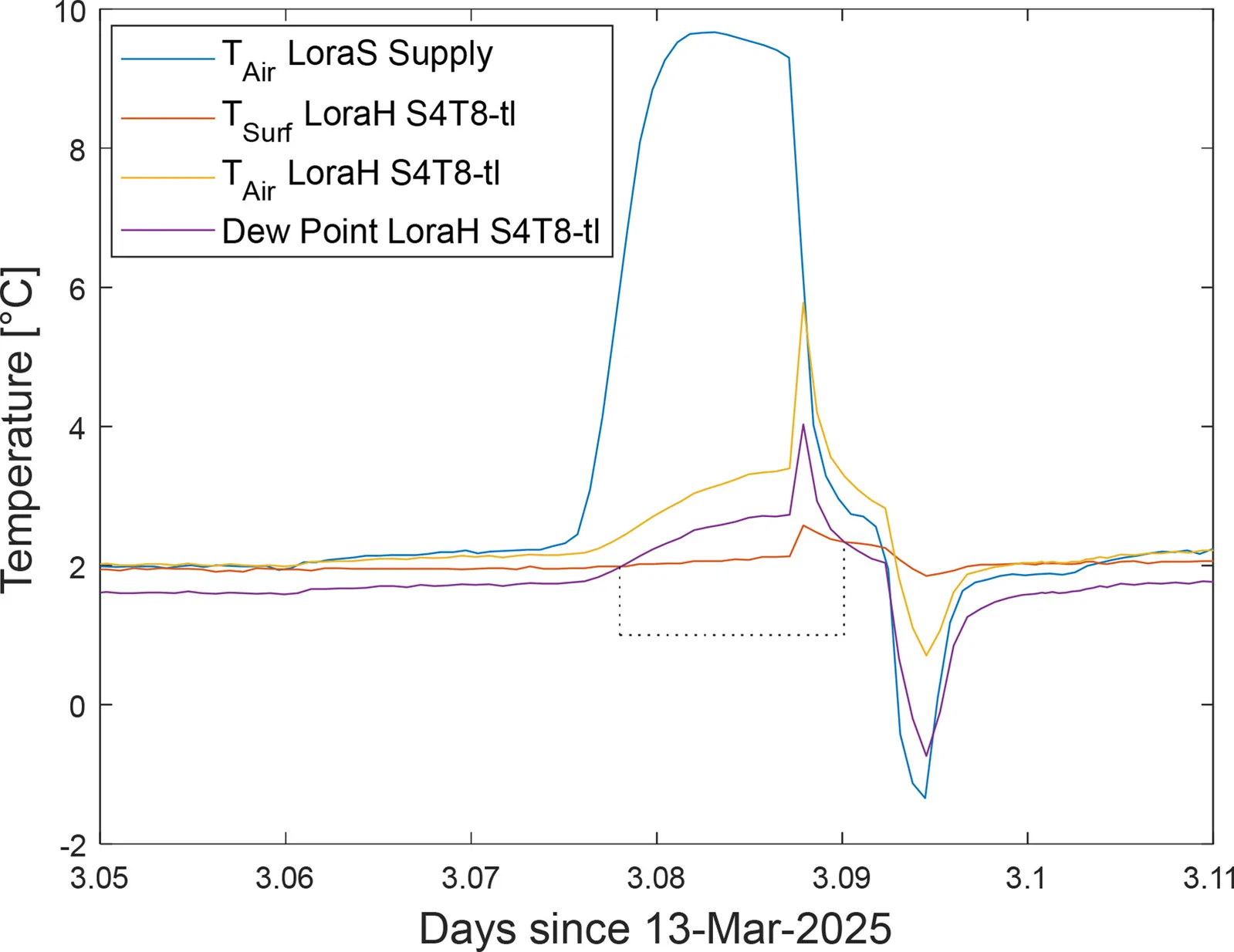
Peak in temperature under the ceiling by electrical defrosting in T4. Surface temperature falls below dew point (manually marked).

The following sample plots for test T4 show the relationship between air, surface and dew point temperature (Fig. 3), variations of humidity (Fig. 4), and an example of the surface temperature falling below the dew point (Fig. 5). The plots were generated by the “plotCombined_T4.m” script with “PLOT_NUM” equal to the figure number.

Example plots for the other tests can be found in [1] or be generated by the MATLAB scripts.

One example script for plotting in Python “plotPythonDemo.py” was added as supplementary file.

### 3.5 Data gaps

As part of quality control, communication gaps were documented in the dataset. Data gaps longer than 4 min are marked by NaN (Not a Number) lines and should be treated as missing values during preprocessing. The NaN lines were used to make data gaps visible in standard plotting tools such as MATLAB and Python. In case a single measurement value has gaps on both sides, only every second gap is marked. NaN lines might be removed by a text editor if not required.

Data gaps of up to 9 h were caused by hang-ups of the sensor node software during T2. The hang-ups were recovered after a watchdog reset every 12 h. Three sensors, which remained for 23 weeks in the cold room, were mostly affected. Sensor D had 13 gaps with a length of > 1 h. During the first 3 weeks of T2, only a single or no gaps > 1 h were observed per sensor. The software hang-ups were fixed for the subsequent tests, which showed no gaps > 1 h.

A counting of missing samples was calculated based on the time difference between an expected but missing sample and the next received sample. Only the first 25 days were evaluated to exclude extended transmission intervals due to low battery conditions. The share of missing samples was <2% for most sensors in T2 except for sensors B, C, and D, which had higher communication losses by deeper positions inside the stack, with losses between 3% and 26%. The losses during the subsequent tests were mostly < 1%, except A, C, D, E, F in T3 (1.5% to 24%), F in T4 (4.6%), and F, G, I in T5 (1.2% to 7%). The positions deep inside the stack showed only slow changes of temperature and humidity, which can be interpolated or reconstructed by signal processing in case of short data gaps. Sensors with losses > 5% were marked in the t*._SensorList.txt files.

Further gaps were caused by failure of the communication gateway over 25 h during T2 on 2024-11-20 and over 1 hour during T5 on 2025-04-01. Shorter gaps were caused by radio communication failures and occasional failures of the I^2^C serial bus for sensor connection.

## 4 Experimental Design, Materials and Methods

The experimental design for recording the temperature and humidity values in a cold room was already described in [1]. This section summarizes the experimental setup of sensors, calibration, data infrastructure, and sensor installation.

### 4.1 Sensors

Wired sensor probes are only feasible in easily accessible bins on the top or at the door side. Positions deeper in the stack require wireless communication by battery powered sensors with a limited energy budget.

Several miniaturized low-power temperature sensors are commercially available with high accuracy. Miniaturized humidity sensors mostly use a capacitive membrane. The membrane absorbs moisture under high humidity conditions over time, leading to a sensor drift, and making careful calibration necessary (see following section). Alternative handheld probes with resistive sensors, e.g. Novasina nSens-HT-EIH rF/T (Switzerland), are almost drift-free, but must be excluded for the cold room tests due to high price, size and energy consumption and can only be applied for reference measurements.

**Fig. 6:**
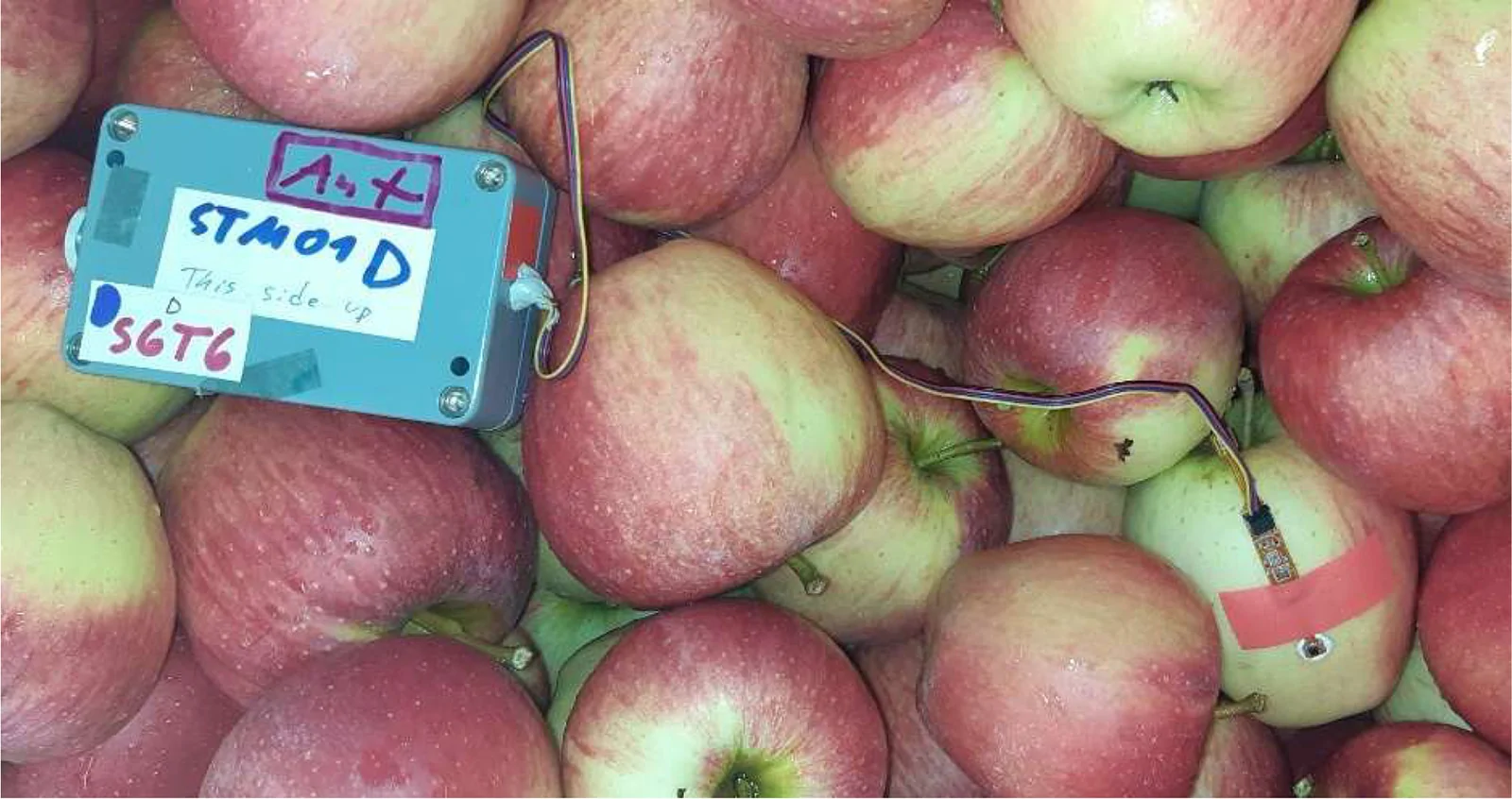
LoRa sensor node and sensor installation in apple bin.

Two chip sensors were mounted on a flexible circuit board of 73 mm length and 0.2 mm thickness. One end was glued on the apple for surface temperature measurement by the Sensirion STS35 sensor. The other end was equipped with a Sensirion SHT45 sensor for air humidity and temperature (Fig. 6).

### 4.2 Calibration

Humidity measurements were affected by the known drift behaviour of capacitive humidity sensors under prolonged high-humidity conditions. Therefore, sensors were calibrated under near-saturated conditions, and the uncertainty estimate includes both calibration error and an additional drift tolerance. The humidity in an air volume above a saturated solution of K_2_SO_4_ was used as a reference. Reference tubes are commercially available, e.g. MHT97-HumdityCheck by Driesen+Kern Germany. At 1.5 °C the tube provides a relative humidity of 98.65% according to [5]. The same principle was applied in our calibration setup, consisting of a jar with K_2_SO_4_ solution, up to six sensor pairs in the airspace above, and an outer box to reduce temperature fluctuations inside a climate chamber. Estimated calibration offsets were subtracted from the sensor data.

Stable humidity values were achieved after 48 h with a remaining drift of 0.5%RH/day. It was assumed that this condition is representative of the long-term expose to high humidity in the cold room. A more detailed discussion of sensor drift and calibration procedures is provided in the related primary research article [1]. Due to the uncertainty of this assumption, a drift tolerance in the magnitude of the drift per one day must be added to measurement errors.

Repeated calibration runs before and after the cold room tests showed an average error of ± 0.03 °C for temperature difference per sensor pair. The humidity calibrations had an average error of ±0.27%RH, see Section 2.1.1. in [1]. Including drift tolerance, a total error below ±1%RH is expected for the humidity measurements.

If condensed water remains on the sensor for longer periods, the membrane becomes fully saturated and values above 100% are displayed, as observed in test T2 for a sensor that remained >4 weeks in the cold room.

### 4.3 Additional sensors

Additional measurements of air speed inside the bins were recorded during test T3. A hotwire anemometer was placed in the centre of four plastic spheres, mimicking the shape of a cluster of apples [6]. The measurements of these air speed loggers (ASL) do not completely fall back to zero air speed when ventilation is switched off due to thermal drift of the sensor and airflow by convection. The ASLs were installed close to the sensor pairs for temperature and humidity measurements. Nearby sensor positions are marked by the same suffix letter of the sensor name.

Furthermore, the CO_2_ and O_2_ measurements of the cooling control system were read out and added to the data set.

### 4.4 Data infrastructure

The sensor data were transmitted wirelessly. Each sensor pair was equipped with a RN2483 radio module for LoRaWAN (Long Range Wide Area Network) communication. Measurements were transmitted in intervals of 60 s or 150 s. Two LoRa gateways were installed under the ceiling and above the door of the cold room. Lost packets due to communication failure are reported in the data set. The gateways forward the data to a public LoRa server, provided by The Things Network (TTN), Netherlands. The Apache Kafka streaming platform was installed on a Linux virtual machine to query the measurements and further processing. Additional wired sensors used a STM32 Nucleo microcontroller for sending measured values to a Raspberry Pi embedded computer via USB in intervals of 30 s. The Raspberry Pi forwarded the data to the Kafka streaming platform. Further details about the data infrastructure can be found in [1] and [7].

### 4.5 Sensor installation

The tests started in a commercial cold room with a new environmentally friendly cooling system with propane as refrigerant and larger evaporators, providing cooling air with less temperature fluctuations. However, the new system did not allow for modifications of cooling parameters. Therefore, the test location was moved to another cold room with a standard controller and R134a as refrigerant.

Cooling on cycle starts when the temperature measured at the wall below the evaporator rises above the higher setpoint and stops when the lower setpoint is achieved (cooling off). This internal sensor is not included in the data set. See Section 2.2 in [1] for details on the cooling control sequence.

Instead, a sensor for return air was mounted at the wall, approximately 1 m below the evaporator. A second sensor for supply air was installed 0.3 m below the ceiling, in approximately 1.5 m distance from the outlet of the evaporator.

Condensed water from the evaporator was channelled to the floor of the cold room.

The typical duration of one cooling interval, i.e. the time between two activations of the cooling unit, is between 0.5 h and 4 h, depending on lower/upper setpoint and on actual fruit temperature. The cooling interval cannot directly be set. Several adjustments of the setpoint values were necessary in test T3 to achieve desired long cooling intervals.

### 4.6 Recommendations for reuse of data and preprocessing

When reusing the dataset, users should consider the practical limitations of commercial cold-room measurements, including communication-related data gaps, occasional extended sampling intervals caused by low battery conditions, sensor drift under high relative humidity, and local variability caused by non-identical sensor placement and airflow paths. Data gaps are explicitly marked in the data files. The first line of the data file contains a comment with the original data set name; the second line contains the header with the names of the columns.

Sensor locations, test conditions, and event logs are provided in the setup and test-specific metadata files to support transparent preprocessing, filtering, and interpretation.

The estimated calibration offsets were already subtracted from the data. The file “CalibrationOffsets.txt” was only included for documentation purposes. Calibration was done after 48 h exposure to high humidity [1]. Measured humidity might be too low during the first day of each test, after the sensors were exposed to lower humidity, while bins were taken outside cold storage to rearrange the storage scheme. The long-term drift under practical cold room conditions was estimated to 1% over 20 days [1].

Relative humidity values above 100% were retained as raw sensor output and were not capped in the dataset or MATLAB plotting scripts. These values should be interpreted as an indication of sensor saturation caused by condensed water on or near the humidity sensor membrane; for quantitative analysis, users should flag these data points and either exclude them or cap them at 100%, depending on the analysis objective.

The experimental data sets contain high numbers of repeated cooling cycles. It is recommended to use cycles without data gaps for further analysis. Otherwise, NaN lines can be skipped. The actual sampling interval can be calculated by the difference of the timestamps in seconds. In case that subsequent processing algorithms cannot handle gaps or require equidistant sampling intervals, resampling of the sensor data [8] should be considered.

If data is interpolated for bins without sensors, or for the left and right row, care must be taken for possibly different initial temperatures. Especially during test T3, bins with sensors were taken out of cooling for some hours for sensor installation, leading to higher fruit temperature.

## Limitations

The data set contains some gaps caused by communication failures. The radio protocol on the sensor nodes occasionally hung up and caused data gaps of max. 9 h during T2. Four sensors switched to extended communication intervals of 10 min or 30 min during T5, due to low battery conditions. The actual sampling interval varies from the default setting by inaccuracies of the micro controller low power clock.

The soldering of two surface sensors broke by mechanical forces and long-term high humidity conditions. Humidity measurements had an error of ±1% due to the known drift behaviour of capacitive humidity sensors under prolonged high-humidity conditions.

All tests used the same basic measurement concept, calibration approach, and data-file format, while the commercial cold-room conditions, sensor positions, atmosphere, and refrigeration settings differed intentionally between tests. This allows comparison across tests while preserving the practical variability of commercial storage conditions.

The limitations of running experiments in commercial cold storage did not allow to control the initial bin temperatures. Depending on the progress of the cooling process, bins had lower initial temperatures for the later tests. Taking out bins from the cold room for sensor installation increased initial temperature variations. Therefore, it was not possible to repeat tests under identical conditions.

Further distorting factors are random in nature: identical positioning of the sensors in the bins was not feasible under practical conditions. Some sensors were more exposed to the air stream than others. Variations in the gap diameter between the stacks also influence the airflow. Measurement data of outside ambient air temperature are available. However, the temperature inside the building depends on factors such as opening of the outside doors, wind, and heating during working hours.

Despite these limitations, the data set provides a good basis for understanding condensation phenomena, spatial distribution of temperature and humidity in the fruit stack, and the effect of modified cooling settings.

## Ethics Statement

The authors confirm to have read and to follow the *ethical requirements* for publication in Data in Brief and confirming that the current work does not involve human subjects, animal experiments, or any data collected from social media platforms.

## CRediT Author Statement

**Reiner Jedermann:** Writing – original draft, Investigation, Software; **Tuany Gabriela Hoffmann:** Writing – review & editing, Investigation; **Daniel Alexandre Neuwald:** Investigation, Resources; **Felix Büchele:** Investigation; **Pramod Vasantrao Mahajan:** Writing – review & editing, Supervision.

## Data Availability

Mendeley DataAir, surface and dew point temperature under high humidity conditions during commercial cold storage of apples (Original data).
